# Supplementary material for: Modeling glioblastoma heterogeneity as a dynamic network of cell states
Source: Mol Syst Biol. 2021 Sep 16;17(9):e10105. doi: 10.15252/msb.202010105 (PMC8444284; doi:10.15252/msb.202010105)
Supplement: Supplementary file 6 — Source Data for Figure 5 [file MSB-17-e10105-s004.zip › Figure5A_sourcedata/GSEA_3017/nosigdb_stateC.GseaPreranked.1622795002785/RICHARDS_NOWAKOWSKI_SCIENCE_2017_VRG_UPREG.html]

Details for gene set RICHARDS\_NOWAKOWSKI\_SCIENCE\_2017\_VRG\_UPREG[GSEA]

|  || Dataset | state13017 |
| Phenotype | NoPhenotypeAvailable |
| Upregulated in class | na\_neg |
| GeneSet | RICHARDS\_NOWAKOWSKI\_SCIENCE\_2017\_VRG\_UPREG |
| Enrichment Score (ES) | -0.4072002 |
| Normalized Enrichment Score (NES) | -2.703725 |
| Nominal p-value | 0.0 |
| FDR q-value | 0.0 |
| FWER p-Value | 0.0 |
Table: GSEA Results Summary

  

Fig 1: Enrichment plot: RICHARDS\_NOWAKOWSKI\_SCIENCE\_2017\_VRG\_UPREG      
 Profile of the Running ES Score & Positions of GeneSet Members on the Rank Ordered List

  

| PROBE | GENE SYMBOL | GENE\_TITLE | RANK IN GENE LIST | RANK METRIC SCORE | RUNNING ES | CORE ENRICHMENT || 1 | SYNE2 |  |  | 0 | 0.839 | 0.0250 | No |
| 2 | MAGI1 |  |  | 6 | 0.652 | 0.0347 | No |
| 3 | ZBTB20 |  |  | 30 | 0.482 | 0.0045 | No |
| 4 | CDCA7L |  |  | 37 | 0.453 | 0.0063 | No |
| 5 | ETV1 |  |  | 44 | 0.438 | 0.0077 | No |
| 6 | PHIP |  |  | 61 | 0.387 | -0.0118 | No |
| 7 | BARD1 |  |  | 86 | 0.357 | -0.0476 | No |
| 8 | CARHSP1 |  |  | 90 | 0.353 | -0.0429 | No |
| 9 | CKB |  |  | 102 | 0.335 | -0.0543 | No |
| 10 | STAG2 |  |  | 128 | 0.309 | -0.0936 | No |
| 11 | SLC12A2 |  |  | 148 | 0.288 | -0.1218 | No |
| 12 | CCND2 |  |  | 149 | 0.288 | -0.1132 | No |
| 13 | KAZN |  |  | 200 | -0.252 | -0.2026 | No |
| 14 | SHC3 |  |  | 206 | -0.253 | -0.2048 | No |
| 15 | FERMT2 |  |  | 227 | -0.259 | -0.2358 | No |
| 16 | SAT1 |  |  | 241 | -0.262 | -0.2532 | No |
| 17 | FDFT1 |  |  | 243 | -0.262 | -0.2473 | No |
| 18 | PCGF5 |  |  | 252 | -0.264 | -0.2550 | No |
| 19 | NPAS3 |  |  | 256 | -0.265 | -0.2529 | No |
| 20 | ID4 |  |  | 261 | -0.267 | -0.2527 | No |
| 21 | GATM |  |  | 272 | -0.270 | -0.2641 | No |
| 22 | HEATR5A |  |  | 278 | -0.271 | -0.2657 | No |
| 23 | DTNA |  |  | 281 | -0.271 | -0.2615 | No |
| 24 | SQLE |  |  | 293 | -0.274 | -0.2747 | No |
| 25 | PHLDA1 |  |  | 295 | -0.274 | -0.2684 | No |
| 26 | SPAG16 |  |  | 300 | -0.277 | -0.2680 | No |
| 27 | SLC1A3 |  |  | 307 | -0.281 | -0.2712 | No |
| 28 | CREB5 |  |  | 309 | -0.281 | -0.2648 | No |
| 29 | LIFR |  |  | 311 | -0.282 | -0.2583 | No |
| 30 | PDPN |  |  | 372 | -0.307 | -0.3655 | No |
| 31 | FOS |  |  | 377 | -0.309 | -0.3640 | No |
| 32 | SAMD4A |  |  | 388 | -0.313 | -0.3741 | No |
| 33 | ZFP36L2 |  |  | 401 | -0.321 | -0.3878 | No |
| 34 | SIPA1L1 |  |  | 412 | -0.329 | -0.3974 | Yes |
| 35 | HMGCR |  |  | 418 | -0.334 | -0.3971 | Yes |
| 36 | LDLR |  |  | 419 | -0.334 | -0.3872 | Yes |
| 37 | CALD1 |  |  | 420 | -0.335 | -0.3772 | Yes |
| 38 | RHOQ |  |  | 426 | -0.338 | -0.3769 | Yes |
| 39 | KLF6 |  |  | 429 | -0.339 | -0.3707 | Yes |
| 40 | REXO2 |  |  | 432 | -0.342 | -0.3644 | Yes |
| 41 | INSIG1 |  |  | 434 | -0.342 | -0.3561 | Yes |
| 42 | FAT1 |  |  | 441 | -0.346 | -0.3574 | Yes |
| 43 | RAB31 |  |  | 445 | -0.349 | -0.3529 | Yes |
| 44 | MYL12A |  |  | 448 | -0.350 | -0.3463 | Yes |
| 45 | BCAN |  |  | 449 | -0.351 | -0.3359 | Yes |
| 46 | IDI1 |  |  | 451 | -0.353 | -0.3273 | Yes |
| 47 | DKK3 |  |  | 454 | -0.356 | -0.3206 | Yes |
| 48 | SEPT9 |  |  | 457 | -0.361 | -0.3137 | Yes |
| 49 | SLITRK2 |  |  | 458 | -0.361 | -0.3030 | Yes |
| 50 | NTRK3 |  |  | 464 | -0.368 | -0.3017 | Yes |
| 51 | HES1 |  |  | 470 | -0.373 | -0.3003 | Yes |
| 52 | LGALS3 |  |  | 471 | -0.374 | -0.2892 | Yes |
| 53 | VCL |  |  | 484 | -0.388 | -0.3009 | Yes |
| 54 | ARL6IP5 |  |  | 490 | -0.396 | -0.2988 | Yes |
| 55 | C1orf61 |  |  | 491 | -0.396 | -0.2870 | Yes |
| 56 | FABP7 |  |  | 495 | -0.411 | -0.2806 | Yes |
| 57 | TGIF1 |  |  | 502 | -0.421 | -0.2797 | Yes |
| 58 | JUN |  |  | 503 | -0.425 | -0.2670 | Yes |
| 59 | SCD |  |  | 504 | -0.431 | -0.2542 | Yes |
| 60 | F3 |  |  | 505 | -0.432 | -0.2413 | Yes |
| 61 | NES |  |  | 509 | -0.435 | -0.2342 | Yes |
| 62 | DBI |  |  | 514 | -0.440 | -0.2289 | Yes |
| 63 | PTN |  |  | 517 | -0.446 | -0.2195 | Yes |
| 64 | CST3 |  |  | 520 | -0.452 | -0.2099 | Yes |
| 65 | MSMO1 |  |  | 525 | -0.460 | -0.2039 | Yes |
| 66 | HMGCS1 |  |  | 529 | -0.466 | -0.1959 | Yes |
| 67 | IFITM3 |  |  | 533 | -0.477 | -0.1875 | Yes |
| 68 | ITGA6 |  |  | 535 | -0.479 | -0.1752 | Yes |
| 69 | TTYH1 |  |  | 540 | -0.496 | -0.1682 | Yes |
| 70 | HOPX |  |  | 546 | -0.513 | -0.1626 | Yes |
| 71 | ANXA2 |  |  | 563 | -0.567 | -0.1767 | Yes |
| 72 | TNC |  |  | 564 | -0.572 | -0.1597 | Yes |
| 73 | MT2A |  |  | 566 | -0.575 | -0.1445 | Yes |
| 74 | FGFBP3 |  |  | 573 | -0.644 | -0.1370 | Yes |
| 75 | GFAP |  |  | 574 | -0.659 | -0.1174 | Yes |
| 76 | VIM |  |  | 578 | -0.674 | -0.1031 | Yes |
| 77 | CLU |  |  | 581 | -0.690 | -0.0865 | Yes |
| 78 | CYR61 |  |  | 584 | -0.714 | -0.0691 | Yes |
| 79 | ATP1B2 |  |  | 585 | -0.714 | -0.0478 | Yes |
| 80 | CA12 |  |  | 586 | -0.717 | -0.0265 | Yes |
| 81 | FABP5 |  |  | 596 | -1.477 | -0.0000 | Yes |
Table: GSEA details [plain text format]

  

Fig 2: RICHARDS\_NOWAKOWSKI\_SCIENCE\_2017\_VRG\_UPREG: Random ES distribution      
 Gene set null distribution of ES for **RICHARDS\_NOWAKOWSKI\_SCIENCE\_2017\_VRG\_UPREG**
